# Supplementary material for: Antitrypanosomal Activity of Sesquiterpene Lactones from Helianthus tuberosus L. Including a New Furanoheliangolide with an Unusual Structure
Source: Molecules. 2019 Mar 18;24(6):1068. doi: 10.3390/molecules24061068 (PMC6471283; doi:10.3390/molecules24061068)
Supplement: Supplementary file 1 [file molecules-24-01068-s001.pdf]

molecules

Supplementary material

Antitrypanosomal Activity of Sesquiterpene Lactones from *Helianthus tuberosus* L. Including a New Furanoheliangolide with an Unusual Structure

Anna Galkina [1], Nico Krause [1], Mairin Lenz [1], Constantin G. Daniliuc [2], Marcel Kaiser [3,4] and Thomas J. Schmidt [1,*]

[1] Institute of Pharmaceutical Biology and Phytochemistry (IPBP), University of Münster, PharmaCampus – Corrensstrasse 48, D-48149 Münster, Germany; no4k-a@yandex.ru (A.G.); Nico1Krause@gmx.de (N.K.); mailen@uni-muenster.de (M.L.)
[2] Institute of Organic Chemistry, University of Münster, Corrensstraße 40, D-48149 Münster, Germany; constantin.daniliuc@uni-muenster.de
[3] Swiss Tropical and Public Health Institute (Swiss TPH), Socinstrasse 57, Basel CH-4051, Switzerland
[4] University of Basel, Petersplatz 1, Basel CH-4003, Switzerland; Email: marcel.kaiser@unibas.ch
* Correspondence: thomschm@uni-muenster.de; Tel.: +49-251-83-33378

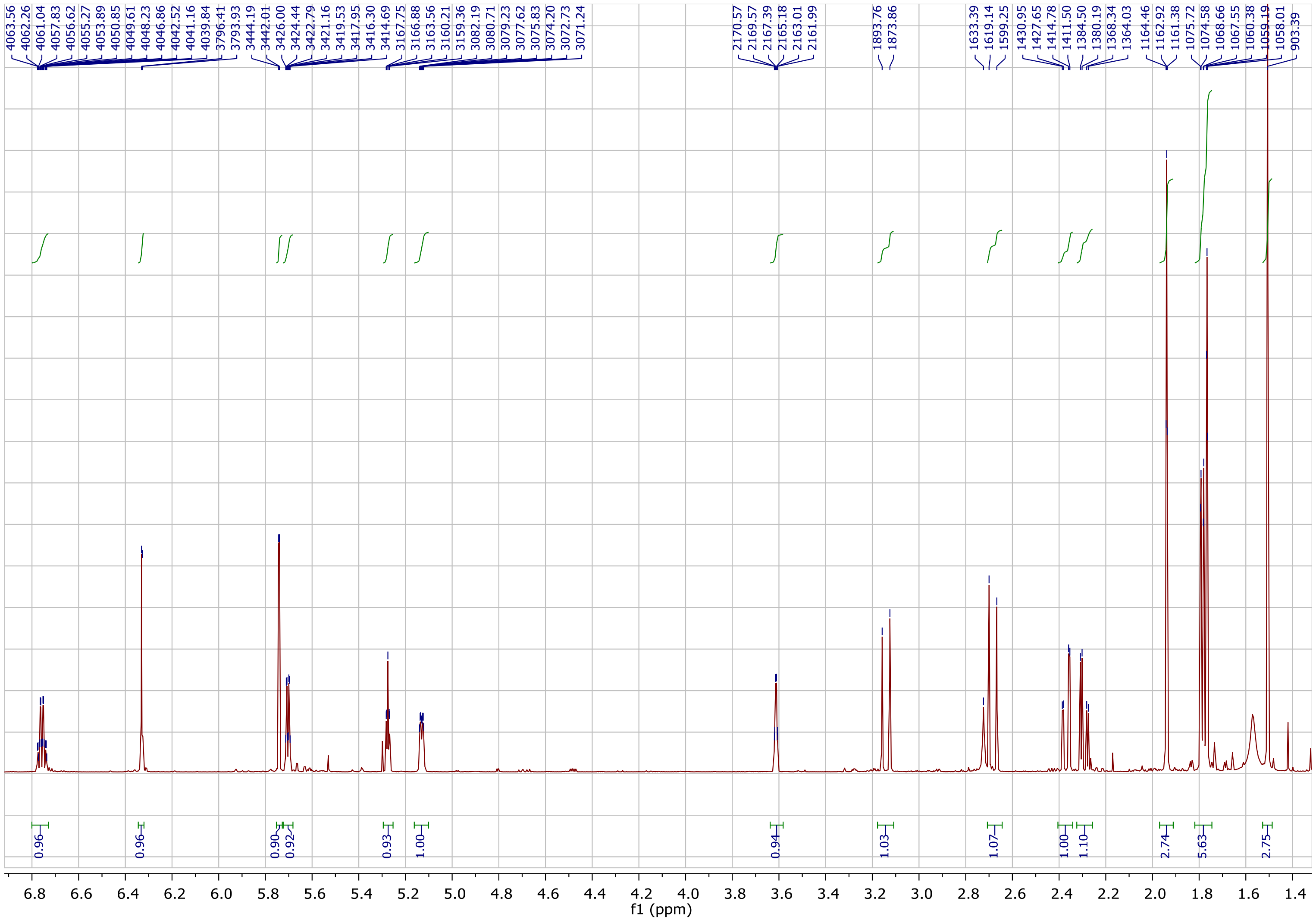

Figure 1. ^{1}H-NMR spectrum of compound **4** (600 MHz, $CDCl_3$).

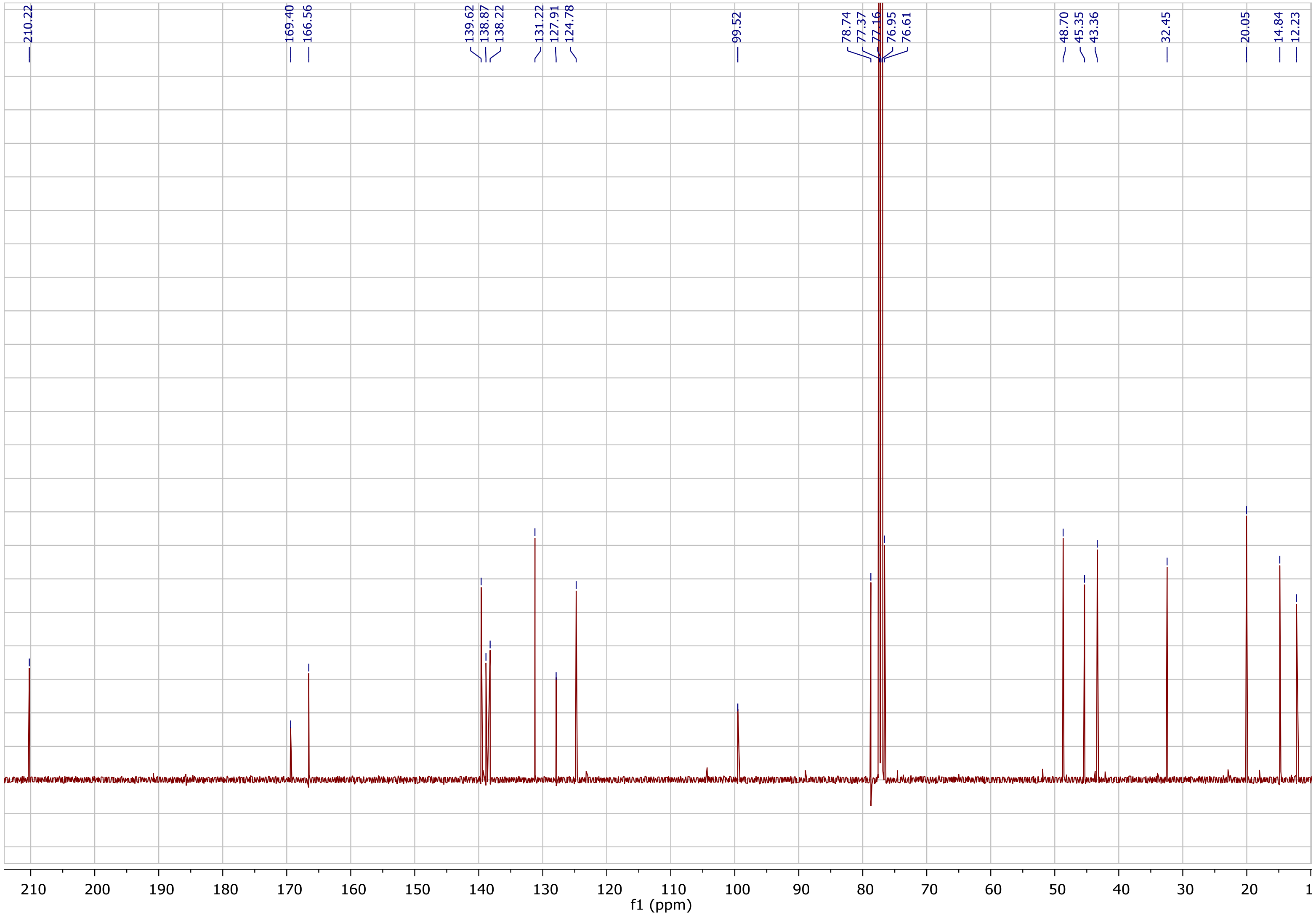

Figure 2. ^{13}C-NMR spectrum of compound **4** (150 MHz, $CDCl_3$).

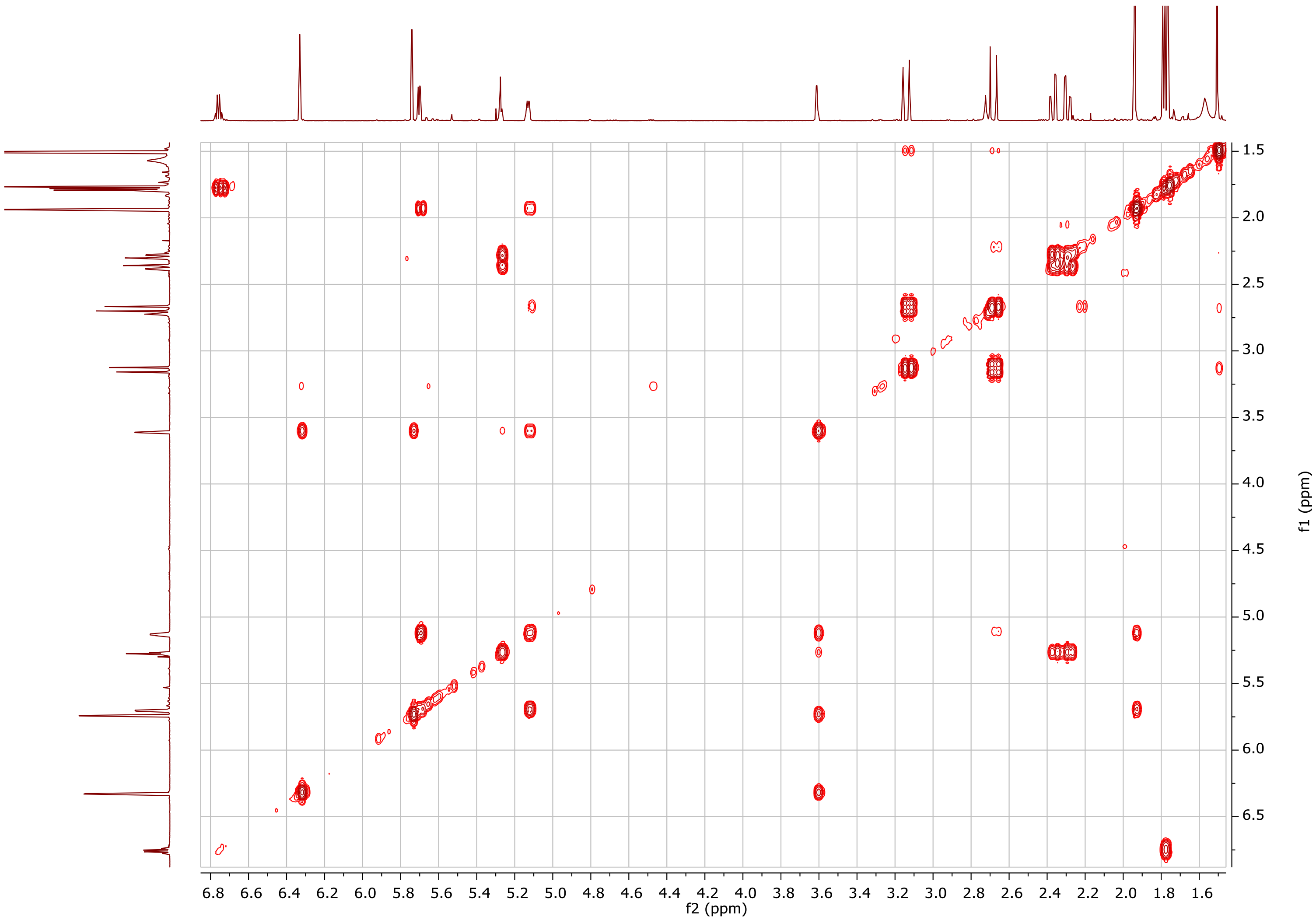

Figure 3. $^1H/^1H$-COSY spectrum of compound **4** (600 MHz, $CDCl_3$).

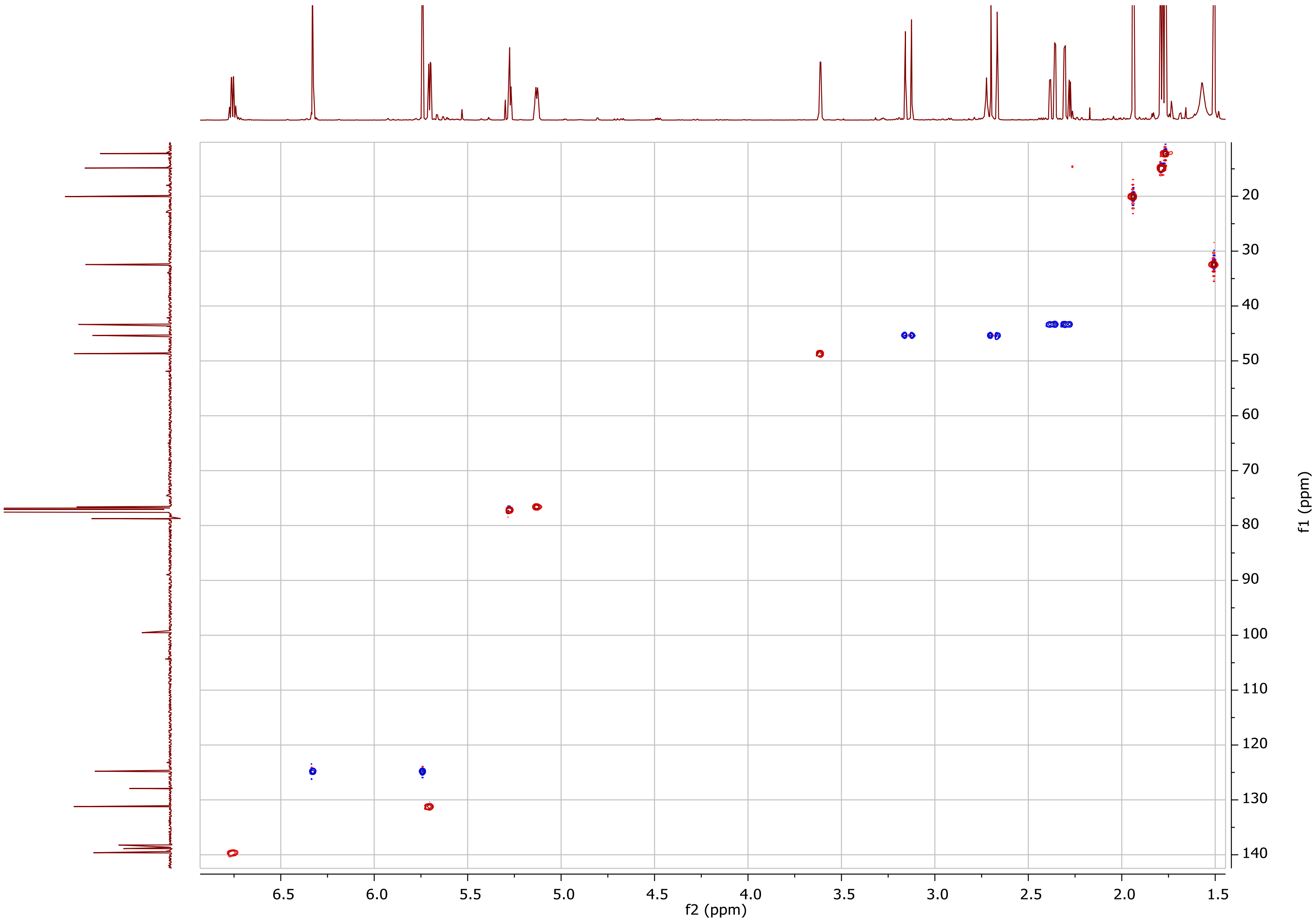

Figure S4. $^{1}H/^{13}C$-HSQC spectrum of compound **4** (600 MHz, $CDCl_3$)

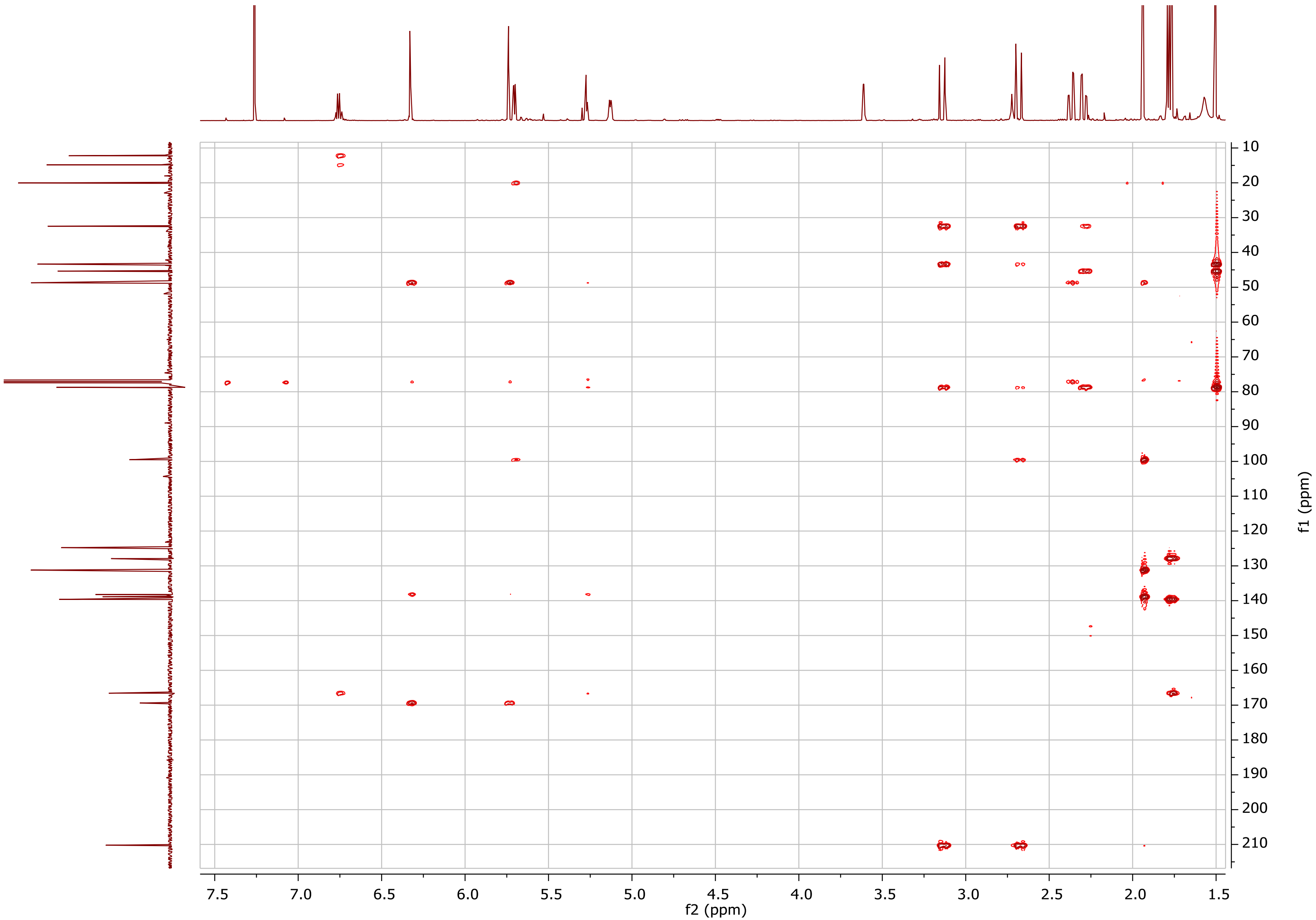

Figure S5. $^{1}H/^{13}C$-HMBC spectrum of compound **4** (600 MHz, $CDCl_3$)

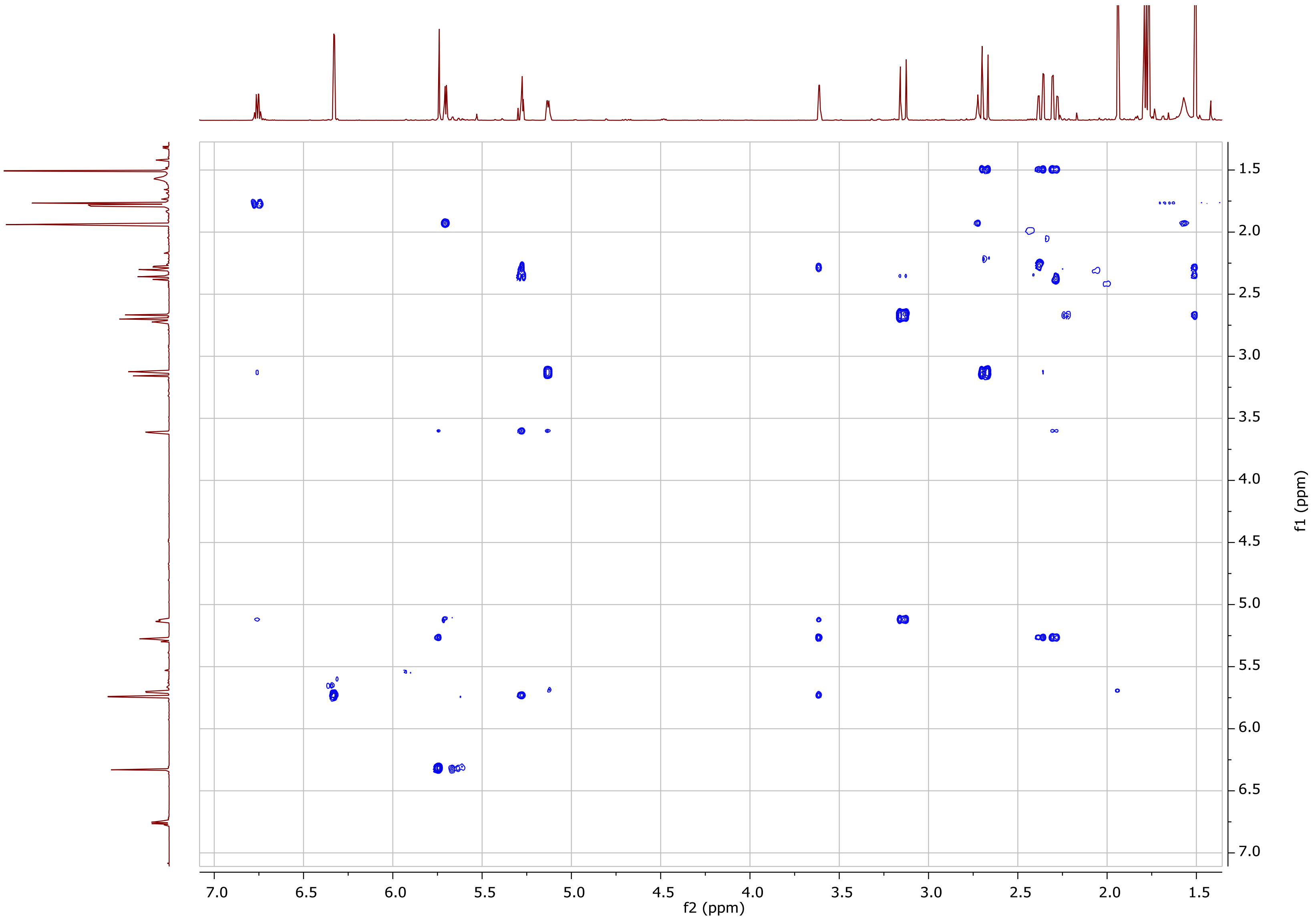

Figure S6. $^1H/^1H$-NOESY spectrum of compound **4** (600 MHz, $CDCl_3$)

Table S1. Data Collection details for dan9339 (compound **4**).

Axis	dx/mm	2θ/°	ω/°	φ/°	χ/°	Width °	Frames	Time/s	Wave-length/Å	Voltage/kV	Current/mA	Temperature/K
Omega	33.971	104.27	-3.89	135.00	62.19	1.10	99	55.00	1.54184	50	1.0	115
Omega	33.971	104.27	108.73	45.00	-44.94	1.10	85	55.00	1.54184	50	1.0	115
Omega	33.971	-2.53	-100.40	90.00	44.94	1.10	85	20.00	1.54184	50	1.0	115
Phi	33.971	104.27	105.54	-163.75	-23.00	1.10	225	40.00	1.54184	50	1.0	115
Phi	33.971	104.27	14.94	-163.65	23.00	1.10	203	55.00	1.54184	50	1.0	115
Omega	33.971	104.27	108.73	90.00	-44.94	1.10	85	55.00	1.54184	50	1.0	115
Phi	33.971	-59.27	40.27	0.15	-44.44	1.10	327	40.00	1.54184	50	1.0	115
Phi	33.971	104.27	107.06	-267.85	-44.44	1.10	247	55.00	1.54184	50	1.0	115

Table S2. Sample and crystal data for dan9339 (compound **4**).

**Identification code**	Dan9339	
**Chemical formula**	$C_{20}H_{24}O_7$	
**Formula weight**	376.39 g/mol	
**Temperature**	115(2) K	
**Wavelength**	1.54178 Å	
**Crystal size**	0.089 × 0.129 × 0.245 mm	
**Crystal habit**	colorless prism	
**Crystal system**	orthorhombic	
**Space group**	P 21 21 21	
**Unit cell dimensions**	a = 7.4900(2) Å	α = 90°
	b = 15.4129(3) Å	β = 90°
	c = 16.6182(4) Å	γ = 90°
**Volume**	1918.45(8) $Å^3$	
**Z**	4	
**Density (calculated)**	1.303 g/cm^3	
**Absorption coefficient**	0.822 mm^{-1}	
**F(000)**	800	

Table S3. Data collection and structure refinement for dan9339 (compound **4**).

**Theta range for data collection**	3.91 to 68.37°	
**Index ranges**	−9 <= h <= 9, −18 <= k <= 18, −19<= l <=20	
**Reflections collected**	18431	
**Independent reflections**	3487 [R(int) = 0.0405]	
**Coverage of independent reflections**	99.7%	
**Absorption correction**	multi-scan	
**Max. and min. transmission**	0.9300 and 0.8240	
**Refinement method**	Full-matrix least-squares on F^2	
**Refinement program**	SHELXL-2014/7 (Sheldrick, 2014)	
**Function minimized**	Σ w($F_o^2 - F_c^2)^2$	
**Data/restraints/parameters**	3487/0/249	
**Goodness-of-fit on F^2**	1.036	
**Final R indices**	3336 data; I > 2σ(I)	R1 = 0.0291, wR2 = 0.0707
	all data	R1 = 0.0309, wR2 = 0.0719
**Weighting scheme**	$w = 1/[\sigma^2(F_o^2) + (0.0415P)^2 + 0.2595P]$; where $P = (F_o^2 + 2F_c^2)/3$	
**Absolute structure parameter**	0.09(6)	
**Largest diff. peak and hole**	0.137 and −0.166 $eÅ^{-3}$	
**R.M.S. deviation from mean**	0.040 $eÅ^{-3}$	

Table S4. Atomic coordinates and equivalent isotropic atomic displacement parameters ($Å^2$) for dan9339 (compound **4**).

	x/a	y/b	z/c	U(eq)
C1	0.5890(2)	0.68732(11)	0.58854(12)	0.0181(4)
C2	0.4686(2)	0.63540(12)	0.53371(12)	0.0174(4)
C3	0.5660(2)	0.54937(12)	0.51482(11)	0.0180(4)
C4	0.4558(2)	0.46653(12)	0.51679(12)	0.0192(4)
C5	0.3689(2)	0.44603(12)	0.58332(12)	0.0204(4)
C6	0.3576(2)	0.50168(12)	0.65717(12)	0.0184(4)

C7	0.5003(2)	0.48677(12)	0.72388(13)	0.0182(4)
C8	0.5968(2)	0.56988(12)	0.75250(12)	0.0183(4)
C9	0.7543(2)	0.60451(12)	0.70338(12)	0.0184(4)
C10	0.7386(2)	0.62444(11)	0.61354(12)	0.0180(4)
C11	0.3903(2)	0.44921(12)	0.79166(12)	0.0209(4)
C12	0.1997(2)	0.45063(13)	0.76815(12)	0.0218(4)
C13	0.4409(3)	0.42247(15)	0.86383(14)	0.0315(5)
C14	0.9200(3)	0.65335(13)	0.58126(13)	0.0242(4)
C15	0.4643(3)	0.40725(13)	0.44509(13)	0.0279(5)
C1'	0.4714(3)	0.69296(12)	0.82249(13)	0.0222(4)
C2'	0.3076(3)	0.74727(12)	0.82807(13)	0.0233(4)
C3'	0.2816(3)	0.79065(12)	0.89667(15)	0.0262(5)
C4'	0.1220(3)	0.84333(14)	0.91915(16)	0.0339(5)
C5'	0.1791(3)	0.74483(17)	0.75874(16)	0.0390(6)
O1	0.32615(17)	0.65711(9)	0.50661(9)	0.0232(3)
O2	0.69368(16)	0.54140(8)	0.57548(8)	0.0181(3)
O3	0.65374(17)	0.56413(9)	0.44056(8)	0.0223(3)
O4	0.45614(16)	0.63448(8)	0.76177(8)	0.0185(3)
O5	0.6010(2)	0.69724(11)	0.86561(10)	0.0347(4)
O6	0.06930(19)	0.42746(10)	0.80546(10)	0.0300(4)
O7	0.18331(16)	0.48419(9)	0.69406(9)	0.0221(3)

U(eq) is defined as one third of the trace of the orthogonalized U_{ii} tensor.

Å) for dan9339 (compound **4**).

C1–C10	1.512(3)	C1–C10	1.538(2)
C1–H1A	0.99	C1–H1B	0.99
C2–O1	1.205(2)	C2–C3	1.546(3)
C3–O2	1.395(2)	C3–O3	1.416(2)
C3–C4	1.521(3)	C4–C5	1.321(3)
C4–C15	1.503(3)	C5–C6	1.500(3)
C5–H5	0.95	C6–O7	1.467(2)
C6–C7	1.557(3)	C6–H6	1.0
C7–C11	1.510(3)	C7–C8	1.546(2)
C7–H7	1.0	C8–O4	1.458(2)
C8–C9	1.531(3)	C8–H8	1.0
C9–C10	1.529(3)	C9–H9A	0.99
C9–H9B	0.99	C10–O2	1.467(2)
C10–C14	1.527(3)	C11–C13	1.324(3)
C11–C12	1.480(3)	C12–O6	1.211(2)
C12–O7	1.341(3)	C13–H13A	0.95
C13–H13B	0.95	C14–H14A	0.98
C14–H14B	0.98	C14–H14C	0.98
C15–H15A	0.98	C15–H15B	0.98
C15–H15C	0.98	C1'–O5	1.209(3)
C1′–O4	1.358(2)	C1'–C2'	1.488(3)
C2′–C3	1.336(3)	C2'–C5'	1.502(3)
C3′–C4′	1.493(3)	C3'–H3	0.95
C4′–H4A	0.98	C4'–H4B	0.98
C4′–H4C	0.98	C5'–H5A	0.98
C5′–H5B	0.98	C5'–H5C	0.98
O3–H3A	0.84		

Table S6. Bond angles (°) for dan9339 (compound **4**).

C2-C1-C10	105.31(14)	C2-C1-H1A	110.7
C10-C1-H1A	110.7	C2-C1-H1B	110.7
C10-C1-H1B	110.7	H1A-C1-H1B	108.8
O1-C2-C1	127.34(17)	O1-C2-C3	125.46(17)
C1-C2-C3	107.14(15)	O2-C3-O3	109.01(14)
O2-C3-C4	106.42(15)	O3-C3-C4	113.92(16)

O2-C3-C2	104.62(15)	O3-C3-C2	104.97(14)
C4-C3-C2	117.35(14)	C5-C4-C15	122.62(18)
C5-C4-C3	119.09(17)	C15-C4-C3	118.04(17)
C4-C5-C6	125.15(17)	C4-C5-H5	117.4
C6-C5-H5	117.4	O7-C6-C5	106.66(14)
O7-C6-C7	106.62(15)	C5-C6-C7	117.38(15)
O7-C6-H6	108.6	C5-C6-H6	108.6
C7-C6-H6	108.6	C11-C7-C8	110.06(17)
C11-C7-C6	102.32(14)	C8-C7-C6	114.69(15)
C11-C7-H7	109.8	C8-C7-H7	109.8
C6-C7-H7	109.8	O4-C8-C9	112.03(14)
O4-C8-C7	105.10(13)	C9-C8-C7	119.03(16)
O4-C8-H8	106.7	C9-C8-H8	106.7
C7-C8-H8	106.7	C10-C9-C8	122.13(15)
C10-C9-H9A	106.8	C8-C9-H9A	106.8
C10-C9-H9B	106.8	C8-C9-H9B	106.8
H9A-C9-H9B	106.6	O2-C10-C14	107.90(15)
O2-C10-C9	105.27(14)	C14-C10-C9	109.47(16)
O2-C10-C1	105.43(14)	C14-C10-C1	111.67(15)
C9-C10-C1	116.50(16)	C13-C11-C12	121.30(19)
C13-C11-C7	129.72(18)	C12-C11-C7	108.87(16)
O6-C12-O7	120.65(17)	O6-C12-C11	129.69(19)
O7-C12-C11	109.65(16)	C11-C13-H13A	120.0
C11-C13-H13B	120.0	H13A-C13-H13B	120.0
C10-C14-H14A	109.5	C10-C14-H14B	109.5
H14A-C14-H14B	109.5	C10-C14-H14C	109.5
H14A-C14-H14C	109.5	H14B-C14-H14C	109.5
C4-C15-H15A	109.5	C4-C15-H15B	109.5
H15A-C15-H15B	109.5	C4-C15-H15C	109.5
H15A-C15-H15C	109.5	H15B-C15-H15C	109.5
O5-C1'-O4	122.98(18)	O5-C1'-C2'	126.50(19)
O4-C1'-C2'	110.51(16)	C3'-C2'-C1'	117.05(19)
C3'-C2'-C5'	125.03(19)	C1'-C2'-C5'	117.81(18)
C2'-C3'-C4'	127.0(2)	C2'-C3'-H3	116.5
C4'-C3'-H3	116.5	C3'-C4'-H4A	109.5
C3'-C4'-H4B	109.5	H4A-C4'-H4B	109.5
C3'-C4'-H4C	109.5	H4A-C4'-H4C	109.5
H4B-C4'-H4C	109.5	C2'-C5'-H5A	109.5
C2'-C5'-H5B	109.5	H5A-C5'-H5B	109.5
C2'-C5'-H5C	109.5	H5A-C5'-H5C	109.5
H5B-C5'-H5C	109.5	C3-O2-C10	113.08(13)
C3-O3-H3A	109.5	C1'-O4-C8	118.11(15)
C12-O7-C6	111.89(14)		

Table S7. Torsion angles (°) for dan9339 (compound **4**).

C10-C1-C2-O1	172.44(19)	C10-C1-C2-C3	−10.20(19)
O1-C2-C3-O2	−163.27(18)	C1-C2-C3-O2	19.31(19)
O1-C2-C3-O3	82.0(2)	C1-C2-C3-O3	−95.42(17)
O1-C2-C3-C4	−45.6(3)	C1-C2-C3-C4	136.94(17)
O2-C3-C4-C5	59.5(2)	O3-C3-C4-C5	179.64(17)
C2-C3-C4-C5	−57.2(2)	O2-C3-C4-C15	−114.96(18)
O3-C3-C4-C15	5.2(2)	C2-C3-C4-C15	128.38(19)
C15-C4-C5-C6	179.06(18)	C3-C4-C5-C6	4.9(3)
C4-C5-C6-O7	148.34(18)	C4-C5-C6-C7	−92.2(2)
O7-C6-C7-C11	7.55(18)	C5-C6-C7-C11	−111.89(17)
O7-C6-C7-C8	−111.59(16)	C5-C6-C7-C8	128.97(18)
C11-C7-C8-O4	−70.66(18)	C6-C7-C8-O4	44.1(2)
C11-C7-C8-C9	162.89(15)	C6-C7-C8-C9	−82.4(2)
O4-C8-C9-C10	−68.4(2)	C7-C8-C9-C10	54.7(2)
C8-C9-C10-O2	−61.4(2)	C8-C9-C10-C14	−177.14(16)
C8-C9-C10-C1	55.0(2)	C2-C1-C10-O2	−2.09(19)
C2-C1-C10-C14	114.83(18)	C2-C1-C10-C9	−118.38(17)
C8-C7-C11-C13	−58.8(3)	C6-C7-C11-C13	178.8(2)
C8-C7-C11-C12	117.40(17)	C6-C7-C11-C12	−4.9(2)

C13-C11-C12-O6	−2.5(4)	C7-C11-C12-O6	−179.1(2)
C13-C11-C12-O7	176.98(19)	C7-C11-C12-O7	0.4(2)
O5-C1'-C2'-C3'	−16.4(3)	O4-C1'-C2'-C3'	162.35(17)
O5-C1'-C2'-C5'	167.3(2)	O4-C1'-C2'-C5'	−13.9(3)
C1'-C2'-C3'-C4'	−174.79(19)	C5'-C2'-C3'-C4'	1.2(3)
O3-C3-O2-C10	90.09(17)	C4-C3-O2-C10	−146.64(15)
C2-C3-O2-C10	−21.76(19)	C14-C10-O2-C3	−103.87(17)
C9-C10-O2-C3	139.30(14)	C1-C10-O2-C3	15.6(2)
O5-C1'-O4-C8	4.9(3)	C2'-C1'-O4-C8	−173.95(15)
C9-C8-O4-C1'	−84.6(2)	C7-C8-O4-C1'	144.73(16)
O6-C12-O7-C6	−175.56(18)	C11-C12-O7-C6	4.9(2)
C5-C6-O7-C12	118.10(16)	C7-C6-O7-C12	−8.1(2)

Anisotropic atomic displacement parameters (Å^2) for dan9339 (compound **4**).

	U_{11}	U_{22}	U_{33}	U_{23}	U_{13}	U_{12}
C1	0.0184(9)	0.0159(8)	0.0200(10)	0.0016(7)	−0.0019(7)	−0.0014(7)
C2	0.0168(9)	0.0182(8)	0.0173(10)	0.0042(7)	0.0017(8)	−0.0028(7)
C3	0.0173(8)	0.0213(9)	0.0153(9)	0.0005(8)	−0.0018(7)	0.0003(7)
C4	0.0194(8)	0.0178(8)	0.0205(10)	−0.0016(7)	−0.0041(8)	−0.0005(7)
C5	0.0190(8)	0.0189(8)	0.0232(11)	0.0004(8)	−0.0033(8)	−0.0040(7)
C6	0.0162(8)	0.0195(8)	0.0194(10)	0.0028(8)	0.0021(7)	−0.0023(7)
C7	0.0178(8)	0.0182(9)	0.0186(10)	0.0020(7)	−0.0006(8)	−0.0003(7)
C8	0.0158(8)	0.0201(9)	0.0189(10)	0.0011(7)	−0.0021(7)	0.0010(7)
C9	0.0134(7)	0.0218(9)	0.0199(11)	−0.0002(8)	−0.0024(7)	−0.0017(7)
C10	0.0161(8)	0.0172(8)	0.0208(10)	0.0001(8)	−0.0019(8)	−0.0020(7)
C11	0.0211(9)	0.0190(9)	0.0227(11)	0.0031(8)	0.0016(7)	−0.0009(7)
C12	0.0211(9)	0.0225(9)	0.0217(11)	−0.0038(8)	0.0015(8)	−0.0021(7)
C13	0.0243(9)	0.0414(12)	0.0290(13)	0.0141(10)	−0.0010(9)	−0.0031(9)
C14	0.0186(9)	0.0280(10)	0.0262(11)	0.0038(8)	0.0014(8)	−0.0036(7)
C15	0.0405(11)	0.0228(9)	0.0204(11)	−0.0041(8)	−0.0014(10)	−0.0032(9)
C1'	0.0229(9)	0.0213(9)	0.0224(11)	−0.0021(8)	−0.0013(8)	−0.0045(7)
C2'	0.0226(9)	0.0191(8)	0.0282(12)	−0.0001(8)	0.0004(8)	−0.0032(8)
C3'	0.0276(10)	0.0188(9)	0.0322(12)	−0.0010(9)	0.0018(9)	−0.0041(7)
C4'	0.0355(12)	0.0234(10)	0.0429(15)	−0.0080(10)	0.0101(11)	−0.0015(8)
C5'	0.0320(11)	0.0429(13)	0.0419(15)	−0.0117(12)	−0.0122(11)	0.0149(10)
O1	0.0184(6)	0.0233(7)	0.0278(8)	0.0034(6)	−0.0044(6)	0.0008(5)
O2	0.0176(6)	0.0177(6)	0.0189(7)	−0.0008(5)	−0.0035(5)	0.0006(5)
O3	0.0191(6)	0.0317(7)	0.0162(7)	0.0002(6)	0.0011(5)	−0.0024(6)
O4	0.0165(6)	0.0200(6)	0.0190(7)	−0.0019(5)	−0.0017(5)	0.0011(5)
O5	0.0280(8)	0.0415(9)	0.0347(10)	−0.0154(7)	−0.0111(7)	0.0048(6)
O6	0.0235(7)	0.0434(8)	0.0231(8)	−0.0025(7)	0.0052(6)	−0.0096(6)

Hydrogen atomic coordinates and isotropic atomic displacement parameters (Å^2) for dan9339 (compound **4**).

	x/a	y/b	z/c	U(eq)
H1A	0.5225	0.7080	0.6363	0.022
H1B	0.6390	0.7381	0.5598	0.022
H5	0.3088	0.3918	0.5845	0.024
H6	0.3616	0.5640	0.6404	0.022
H7	0.5899	0.4431	0.7052	0.022
H8	0.6435	0.5574	0.8077	0.022
H9A	0.7942	0.6586	0.7299	0.022
H9B	0.8526	0.5621	0.7094	0.022
H13A	0.3541	0.4037	0.9017	0.038
H13B	0.5639	0.4221	0.8777	0.038
H14A	1.0093	0.6085	0.5926	0.036
H14B	0.9553	0.7077	0.6074	0.036
H14C	0.9119	0.6624	0.5230	0.036
H15A	0.3978	0.3539	0.4566	0.042
H15B	0.5892	0.3928	0.4336	0.042
H15C	0.4116	0.4363	0.3983	0.042
H3	0.3748	0.7877	0.9353	0.031
H4A	0.1543	0.9049	0.9202	0.051
H4B	0.0797	0.8256	0.9725	0.051

H4C	0.0270	0.8340	0.8795	0.051
H5A	0.1285	0.6864	0.7539	0.058
H5B	0.2420	0.7598	0.7090	0.058
H5C	0.0827	0.7867	0.7681	0.058
H3A	0.5778	0.5669	0.4035	0.033

Table S10. Hydrogen bond distances (Å) and angles (°) for dan9339 (compound **4**).

	**Donor-H**	**Acceptor-H**	**Donor-Acceptor**	**Angle**
C1-H1B···O1	0.99	2.41	3.377(2)	166.3
O3-H3A···O6	0.84	1.97	2.801(2)	171.4
